# Activation of dorsolateral prefrontal cortex in a dual neuropsychological screening test: An fMRI approach

**DOI:** 10.1186/1744-9081-8-26

**Published:** 2012-05-28

**Authors:** Atsumichi Tachibana, J Adam Noah, Shaw Bronner, Yumie Ono, Yoshiyuki Hirano, Masami Niwa, Kazuko Watanabe, Minoru Onozuka

**Affiliations:** 1 Departments of Physiology & Neuroscience, Kanagawa Dental College, Yokosuka, Kanagawa, Japan; 2 Research Center of Brain & Oral Sciences, Kanagawa Dental College, Yokosuka, Kanagawa, Japan; 3 ADAM Center, Long Island University, Brooklyn, NY, USA; 4 Department of Electronics and Bioinformatics, School of Science and Technology, Meiji University, Kawasaki, Kanagawa, Japan; 5 Research Center for Child Mental Development, Chiba University Graduate School of Medicine, Chiba, Chiba, Japan; 6 Department of Radiology, Tohno Welfare Hospital, Mizunami, Japan; 7 Faculty of Care and Rehabilitation (Physiology), Seijoh University, Tohkai, Aichi, Japan

**Keywords:** Executive function, Neuropsychological test, Working memory

## Abstract

**Background:**

The *Kana Pick-out Test* (KPT), which uses *Kana or* Japanese symbols that represent syllables, requires parallel processing of discrete (pick-out) and continuous (reading) dual tasks. As a dual task, the KPT is thought to test working memory and executive function, particularly in the prefrontal cortex (PFC), and is widely used in Japan as a clinical screen for dementia. Nevertheless, there has been little neurological investigation into PFC activity during this test.

**Methods:**

We used functional magnetic resonance imaging (fMRI) to evaluate changes in the blood oxygenation level-dependent (BOLD) signal in young healthy adults during performance of a computerized KPT dual task (comprised of reading comprehension and picking out vowels) and compared it to its single task components (reading or vowel pick-out alone).

**Results:**

Behavioral performance of the KPT degraded compared to its single task components. Performance of the KPT markedly increased BOLD signal intensity in the PFC, and also activated sensorimotor, parietal association, and visual cortex areas. In conjunction analyses, bilateral BOLD signal in the dorsolateral PFC (Brodmann's areas 45, 46) was present only in the KPT.

**Conclusions:**

Our results support the central bottleneck theory and suggest that the dorsolateral PFC is an important mediator of neural activity for both short-term storage and executive processes. Quantitative evaluation of the KPT with fMRI in healthy adults is the first step towards understanding the effects of aging or cognitive impairment on KPT performance.

## Background

Working memory is thought to be comprised of two components: temporary storage (e.g. short-term memory) and executive processes [1]. Both components contribute to higher cognitive functions such as comprehension, reasoning, planning, problem solving, and learning, which are mediated by the prefrontal cortex (PFC) [2,3]. The dual task paradigm, performance of two tasks concurrently, is often used to study working memory. Diminished performance during dual tasks, compared to performance of separate single tasks, is attributed to the allocation of limited resources to attend to and perform competing task requirements. A meta-analysis of neuro-imaging studies of working memory suggests that tasks of working memory differ in several ways and may, therefore, produce neural activity in different cortical regions [4]. These include types of executive function processing (e.g. continuous updating, temporal order, or manipulation of information) and types of storage (e.g. spatial, verbal, or object). Several mechanisms have been proposed to explain dual task performance decrements. One theory, the central bottleneck, suggests that there is an amodal ‘unified’ processing bottleneck slowing response due to competition and interference by the two tasks, regardless of executive function processing or type of storage [5,6]. Research supports the existance of a common unified neural bottleneck for dual tasks, located in the PFC [5].

The *Kana Pick-out Test* (KPT) was developed as a dual task tool to test cognitive function in persons with dementia and is widely used in Japan to screen for mild cognitive impairment (MCI) [7]. More recently, it has been used to test executive function in children and adolescents with fatigue [8-10]. In the KPT, subjects read a short story and, while reading, circle characters that comprise five Japanese vowels (a, i, u, e, o) within a limited time. Kana is a phoenetic alphabet consisting of pictorial shape-letters that represent sounds or syllables. Immediately, after picking-out the vowels, subjects are asked to answer questions related to the story. Based on data from 20,000 healthy subjects, KPT performance peaks were detected in young healthy adults with a linear decline after the age of 30 [7]. Recently, a computerized version of the test was developed for mass screening [11]. Additionally, the Color Word Pick-out Test was adapted from the KPT as a dementia screening tool for use in other languages [12].

The KPT requires parallel processing of discrete (pick-out) and continuous (reading) dual tasks. As a dual task, the KPT is thought to test executive function, requiring visual perception and information processing, divided attention to pick-out the vowels while understanding the story content, and retrieval and manipulation of stored information for recall of the story elements. Performance decrements (e.g. decreased KPT scores) have been correlated to increasing age and to subjects with known lesions of the dorsolateral PFC (dlPFC) [13,14]. However, there has been minimal quantitative evaluation of the KPT with fMRI. Understanding of PFC activity correlated to the KPT in healthy adults is the first step in understanding the dysfunction or the decrement of dual (or multiple) task performance often seen in geriatric conditions such as Alzheimer's disease, Parkinson's disease, or mild cognitive impairment. There is some suggestion that older adults can improve their dual task performance with training [15,16]. With an aging population, it is important to identify and intervene at the early stage of any cognitive deterioration.

Because the KPT is used so broadly in Japan, determining brain activation patterns may be of particular utility for healthy care providers in Japan. To investigate the brain-behavior relationship of the KPT, we compared the dual task, KPT, to single tasks embedded within the KPT: 1) vowel pick-out and 2) reading comprehension. We examined cortical changes in blood oxygenation level-dependent (BOLD) signal using fMRI during performance of the three tests. We hypothesized that PFC activity would be greater in the KPT dual task compared to the two single task KPT components; To substantiate the bottleneck hypothesis, we hypothesized that KPT performance would demonstrate performance decrement compared to single tasks and the KPT dual task neural activity would differ in the PFC from that of single tasks. Researchers have reported that the dlPFC plays a crucial role in the processing required to perform these tasks but activity in this area is not implicated in single tasks [17-21]. Therefore, dlPFC activity during the KPT was of particular interest in this study.

## Methods

### Subjects

Nineteen healthy right-handed subjects (14 males, 5 females, mean *±* SD age; 24.9 ± 4.94 years, range 20–33 years) provided written informed consent prior to participation in this study. All subjects had, at a minimum, a high school diploma. Reading literacy in Japan is 99.9%, therefore, no reading competency tests were administered. According to test norms, subjects in this age group should have optimal performance on the KPT [7].

### Computerized KPT

**Figure 1:**
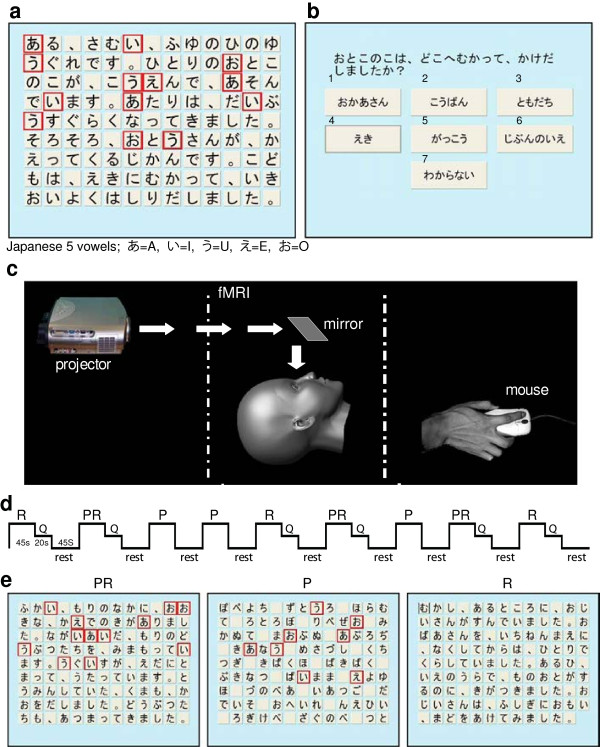
**(a) Computerized KPT.** Selected vowel characters are indicated in red. Literal translation: *It was twilight, close to sunset during a cold winter. A boy was playing in a park. Dusk was the time in which his father usually returned home. So, the boy jumped up and ran to the train station*. **(b)** A question with 7 choices about story content was presented immediately after testing. Literal translation: *Where did the boy run to?* (1) *his mother*, (2) *police box*, (3) *his friend*, (4) *train station*, (5) *his school*, (6) *his home*, (7) *no idea*. **(c)** fMRI set up*.***(d)** Task paradigm. The duration of each block task is 45 sec including the first 5 sec to inform which task to perform (PR, P or R), but the first 5 sec was removed as different regressor when the BOLD signal derived from the task performance was analyzed. **(e)** Tasks. PR (KPT; dual task): picking vowels out of a short story and retaining story content; P (simple task I): picking vowels out of a randomized kana sequence; R (simple task II): reading and retaining a short story without picking vowels out. Note: Q; questions about the story were asked only after PR and R

The computerized KPT [11], provides three original stories, each with 117 Kana characters (containing 18, 20, and 26 vowels). Subjects are asked to read the story on a monitor, retain the story content, and identify as many of the five vowel characters as possible within a limited time (45s) (Figure 1a). If subjects recognize a vowel character, they click the mouse on the character. The square surrounding the character changes color to indicate that the character has been selected. (If the selected character is clicked again, the choice is canceled and it returns to its original color.) The three stories collectively consist of 351 symbols with 64 vowels, so the best possible score for this test is 64. After 45s, the first window disappears and a second window displays a question to examine whether subjects retained the substance of the story (Figure 1b). Each question provides seven answer choices, and subjects are asked to select only one. A second question appears immediately after the first is answered to further test story comprehension. This procedure is repeated for all three stories.

### Protocol

The computerized KPT was reprogrammed to allow us to test both single and dual tasks within the KPT during fMRI data collection (Figure 1c). During testing, subjects lay in supine and viewed the KPT tasks projected onto a screen via a mirror located in the MRI head coil. Subjects held the mouse in their right hand and clicked it when selecting vowels during the reading task. Practice sessions were provided before the experiment, both outside and in the scanner to allow the subjects to become accustomed to the experimental setup.

We employed a sequential task-activation paradigm consisting of 9 blocks for the three tasks presented in randomized order: (1) picking vowels and reading (PR; e.g., KPT dual task), (2) picking vowels only in a randomized Kana sequence with no meaning (P; e.g., single task I), and (3) reading only (R; e.g., single task II) (Figures 1d-e). Therefore, one task was performed in one ‘block’ and tasks were performed a total of three times. For PR, each story utilized the identical text as the one described in the computerized KPT by Inoue et al [11]. For R, we prepared an additional three short stories, each consisting of 117 Kana characters. All of the stories used were similar in content to the original paper-based KPT by Kaneko [7], consisting of 406 Kana characters. We slightly modified the stories to match the 117 characters used by Inoue for consistency. Subjects were informed which task to perform 5s before starting. During rest periods between each session (40s), subjects stared at a tessellated (mosaic) version of the KPT for visual stimulation. [Note: the tessellated version required no response and served as the control contrast.] After PR and R, two questions about the story content were presented (7 answer choices/question/10s) and subjects were asked to select one. Because there were three stories for PR and three stories for R, subjects answered a total of 12 questions (2 questions/story /task). In R, subjects were required to track reading with the mouse. In P, subjects were required only to pickout the vowel characters (i.e. a, i, u, e, o) found in a randomized Kana sequence with no meaning.

Functional MR images, followed by an anatomic (T1 weighted) image, were acquired for each subject using a 1.5-T EXCITE HD MRI scanner (General Electric, Milwaukee, WI). Functional images consisted of echo-planar image (EPI) volumes sensitive to gradient-echo BOLD contrast in the axial orientation (TE = 30 ms, TR = 2500 ms). The volume covered the entire brain with a 64 x 64 matrix and 33 slices (voxel size = 3.75 mm x 3.75 mm x 4 mm, slice thickness = 3.8 mm, gap = 0.2 mm). Images with 580 volumes were acquired.

### Data Analysis

For behavioral analyses, we compared scores of PR versus P and PR versus R using paired t-tests (DOF 18, t = 1.734, p < 0.05).

BOLD signal analyses were performed using SPM8 (The Wellcome Trust Centre for Neuroimaging, London, implemented in MATLAB R2009a (The MathWorks, Inc, Natick, MA, USA; http://www.fil.ion.ucl.ac.uk/spm/). After discarding the first 8 volumes of the EPI series to minimize T2* relaxation artifact, functional data were motion corrected using SPM8 software (Wellcome Department of Cognitive Neurology, London). The realigned images were normalized to the stereotactic coordinate system standard SPM/MNI (Montreal Neurological Institute) template with a 2 mm^3^ resolution, transformed into a standard steorotaxic space, and spatially smoothed using a Gaussian kernel of 8 mm full width at half maximum (FWHM). In all subjects, head motions were less than 0.01 mm in all axes. The data were motion corrected using the default value of 8mm.

Subject-level statistical analyses were performed using the general linear model to create statistical parametric maps. Linear contrasts were constructed to obtain subject-specific estimates for a contrast representing the effect of the active condition (PR, P, or R) compared with rest condition (tessellated KPT) using an activity mask (p < 0.05).

To make broader group inferences, these estimates were entered into standard SPM second-level random effects analyses. 1) Subtraction contrasts comparing dual to single tasks were performed [e.g., PR *−* R and PR *−* P]. 2) Between condition conjunction analyses (e.g., [PR − R]∩[PR − P]) were performed to show common areas of activation using a minimum statistic compared to the conjunction null (MS/CN) [22].

Group results were computed as t-tests in order to derive statistical parametric maps (SPMs) of the normal Z-distribution, identifying brain activity for each condition with a Family-wise Error rate of p < 0.05. Multiple linear regressions were used to identify voxels that correlated with 1) contrasts across conditions and 2) conjunction, also with a Family-wise Error rate of p < 0.05. Anatomical regions were identified using the WFU PickAtlas tool [23,24] with the PFC (e.g. Brodmann's areas: BA9, 10, 11, 44, 45, 46, and 47) selected as the region of interest.

## Results

### Behavioral results

**Figure 2:**
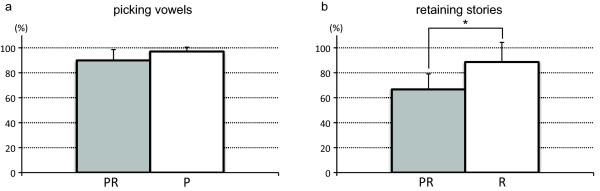
**Comparison of (a) score (percent correct) obtained from dual task (PR; KPT) and simple task I (P), and (b) score (percent correct) obtained from dual task (PR; KPT) and simple task II (R).** Each column represents mean ± SE. *p < 0.05.

In PR (KPT), the mean score of correct vowels was 54.84/61 or 89.9% (Figure 2a). In P, the mean score of correct vowels was 59.17/61 or 97.0%. Vowel-selection score for PR (KPT) was lower than that in P (p < 0.05), Mean percentage of correct answers for questions about story content was lower in PR (66.7%) compared to R (88.6%) (p < 0.05) (Figure 2b).

### *Regional cortical activity* of *task minus rest*

**Figure 3:**
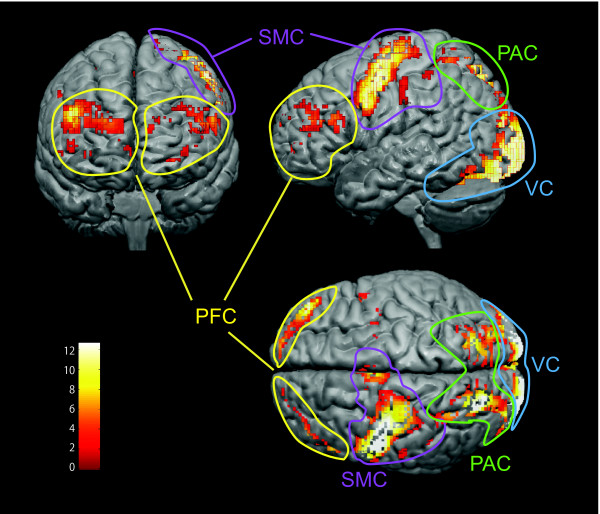
**Regional brain activity during performance of the computerized KPT (e.g., PR).** Activated areas were superimposed on a normalized brain (p < 0.05), corrected. Abbreviations, PFC; prefrontal cortex (yellow circles), SMC; sensorimotor cortex (purple circle), VC; visual cortex (blue circle); PAC, parietal association cortex (green circle). The color scale bars indicating the Z value ranges.

Group analyses of the PR task vs. rest contrast revealed increases in BOLD signal intensity bilaterally in the PFC, (BA 8, 9, 10, 11, 45, 46 and 47) (Figure 3, Table 1). In addition, activity was observed in the left sensorimotor cortex (SMC) including supplementary and pre-motor areas (BA 1, 2, 3, 4 and 6), bilateral parietal association cortex (PAC) (BA 7 and 40), and bilaterally in the primary and inferior temporal visual cortex (VC) during performance of the KPT (PR).

**Table 1:** Prefrontal cortex activation

**Task**	**Hemispheres**	**Brain region**	**Cluster size (Voxels)**	**MNI coordinates**			**Peak t-value**
				**x**	**y**	**z**	
PR	Left	BA 8, 9, 10,11, 45, 46	149	-54	30	24	3.6459
	Right	BA 9, 10, 11, 46, 47	225	46	48	24	5.9596
				Total		374				
P	Left	BA 8, 9, 10, 45, 46	103	-26	58	-6	6.8946		
	Right	BA 8, 9, 10, 11, 46, 47	273	46	48	24	4.0827		
				Total		366				
R	Left	BA 8, 9, 10, 45,46, 47	10	-16	64	24	1.9442		
	Right	BA 9, 10, 45, 46	19	58	30	6	2.186		
	Total		29						

Statistical threshold was set at p < 0.05 (corrected for multiple comparisons).

*PR*: dual task (Kana Pick-out Test), *P*: single task I (picking vowels only), *R*: single task II (reading only).

### Region of interest activity during dual and single tasks

**Figure 4:**
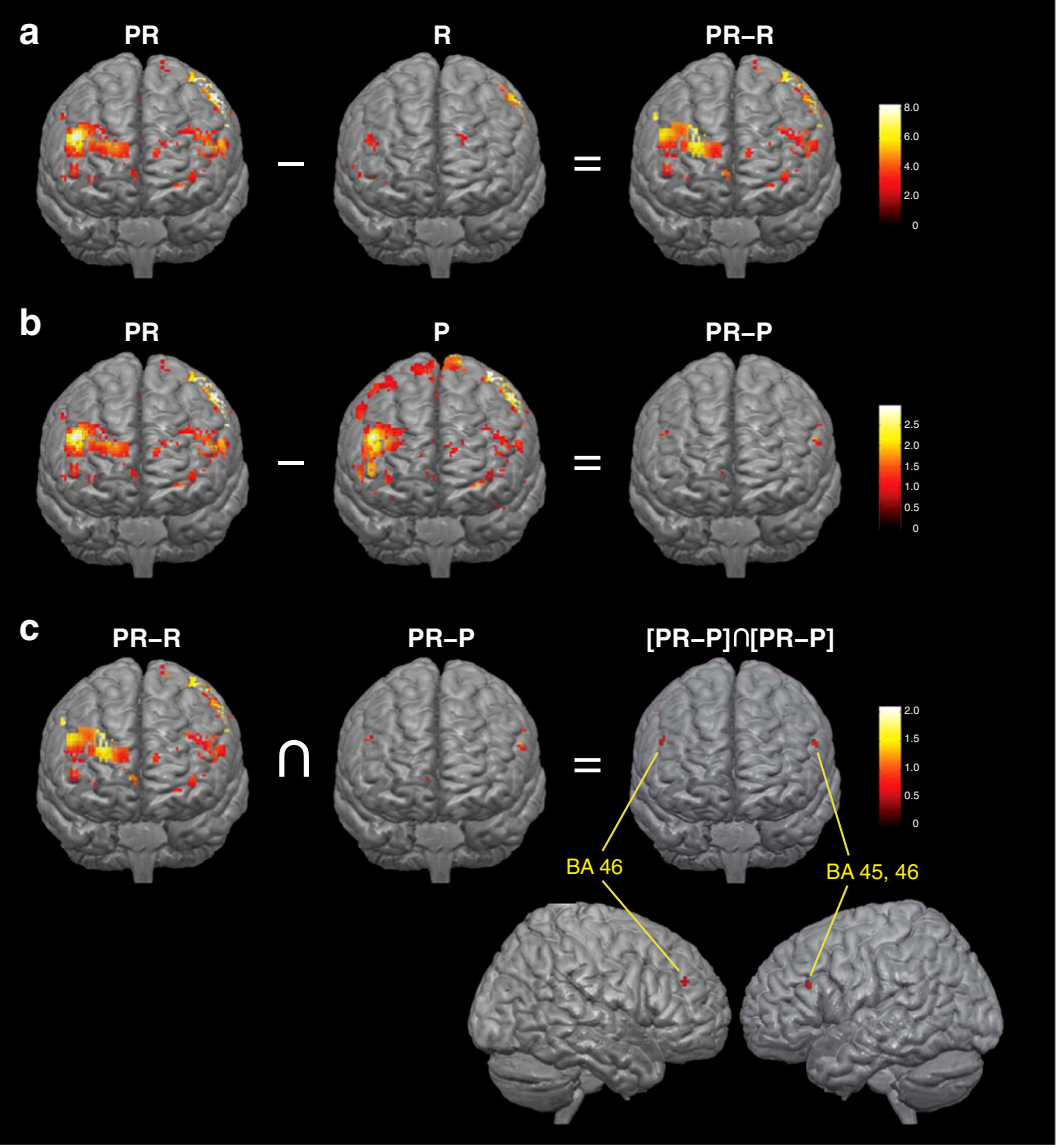
**(a) Regional brain activity during performance of PR, R, and the contrast of PR − P.** (**b**) Regional brain activity during performance of PR, P, and the contrast of PR − R. (**c**) Conjunction analysis from results of contrast analyses (a) and (b) e.g. [PR *−* R]∩[PR *−* P]. (p < 0.05). The color scale bars indicating the Z value ranges.

Increases in BOLD signal intensity in the PFC were observed not only during performance of the dual task (PR), but also during performance of the single tasks (P and R) (Figure 4a and b, Table 1). In R, the BOLD signal cluster size in bilateral PFC was smaller (29 voxels) than that in PR (374 voxels). Activated regions decreased by 6.7% and 8.4% in left and right PFC, respectively. In P, cluster size (366 voxels) was similar to that in PR. Maximal t values in R were lower than in PR. Maximal t values in P were higher in the left PFC and lower in the right PCF than those in PR respectively.

To detect regional activation clusters attributed to dual task processing, independent contrasts were first calculated (e.g., [PR − R] and [PR − P]) (Figures 4a-b). In the second step, only areas active in both contrasts were considered, since such conjunct areas were specifically related to the dual task component (e.g., [PR − R]∩[PR − P]) (Figure 4c) [25]. Using this approach, regional activation clusters were detected only in dorsolateral PFC (dlPFC) bilaterally (BA 45, 46) (Table 2).

**Table 2:** **Conjunction analysis [PR*−*R]∩[PR*−*P]**

**Cluster**	**Hemispheres**	**Brain region**	**Voxels**	**MNI coordinates**			**Peak t-value**
				**x**	**y**	**z**	
1	Left	BA 45	5	-56	26	24	2.0821
		BA 46					
2	Right	BA 46	5	48	44	28	2.0821

Statistical threshold was set at p < 0.05 (corrected for multiple comparisons).

## Discussion

Neuro-imaging studies of dual tasks generally report two types of effects. First, there is degradation of performance in one or both tasks relative to performance in either single task alone. Second, there is increased activation in the PFC in the dual task compared to each single task [5,21,26,27]. Our results support the central bottleneck theory and suggest that the dlPFC is an important mediator of neural activity for both short-term storage and executive processes. To our knowledge, this is the first study to investigate specific behavioral and functional details of regional brain activation during both the dual and single components of the KPT.

### Behavioral performance

In the neuropsychological field, the KPT is administered as a dual task (PR) and compared to the single tasks (P or R). The percentage of correct answers regarding reading content and picked vowels in PR decreased more than the percentage of correct story answers in R and percentage of picked vowels in P respectively. Therefore, dual task performance was diminished for both continuous and discrete task components of the KPT.

Scores obtained for picking vowels in over 20,000 people have been clearly described [7], but there is currently a lack of data about story substance scores. Comparison of our behavioral results of the KPT to those previously reported revealed similar vowel scores [7,14,28]. In our reading content results, PR reading scores were 22% lower than R scores. The variance in the reading component of R was similar to that in PR (Figure 2b). This suggests that the 22% drop in reading performance in PR was primarily due to the addition of the vowel pickout.

During these tasks, the maximal increase in BOLD signal in PFC in PR was greater than that in R. The lower reading scores in PR compared to R and greater PFC activity during PR compared to R suggests that during the PR condition there was increased dual task difficulty. Alternatively, the PFC BOLD signal cluster size in P was similar to that in PR and the differences in vowel pick-out scores were not significant. This suggests that the P task (picking the vowels out in a randomized Kana sequence) had some task complexity. We attribute this to the difficulty of picking out vowels embeded within a randomized Kana sequence with no meaning. It is easier to identify vowels within a well-known word in which one can predict the location of a given vowel.

### General cortical activity

In Japanese Kana, each symbol represents a syllable or vowel, as opposed to kanji in which each character represents a word with a specific meaning. Similar to the English alphabet, Kana permits phonetic reading. Cortical activity during performance of the KPT was found bilaterally in the PFC, left SMC, bilateral PAC, and bilateral VC. Contralateral SMC in the hand region for right hand manipulation was strongly activated by mouse clicking. This activation is in agreement with SMC activity during finger tapping, finger press, and mouse clicking experiments [29]. Increased VC activity was related to subjects viewing the screen to perform testing and similar to reports of Kana reading [30-32]. Cognitive tasks such as reading demonstrate greater activations in VC than staring at pseudo objects [33]. However, other studies report similar VC activity for reading Kana words and non-words [34].

Imaging studies of Kana reading report that it is primarily processed in the left hemisphere, particularly the left dlPFC, superior and inferior parietal lobe, temporal- parietal area, and posterior inferior temporal gyrus [30-32,34,35]. This is similar to alphabetic reading imaging reports of neural activity in the left PFC and temporal lobe language processing areas (e.g. Broca’s and Wernicke’s areas) [30,33,36].

Activation of the PAC during the KPT was attributed to spatial perception for searching and retrieving vowels with voluntary control of spatial attention for both vision and touch [37]. In particular, the superior parietal lobe has been described as a part of top-down processing and feed-forward modulation of sensory inputs, integrating visual input to execute goal-directed spatial orienting [38]. Activity in the inferior parietal lobule, specifically BA 40, is reported during phonological processing [36].

In some subjects, Wernicke’s area is activated by language processing for retaining the story semantics. In young subjects, increases in Wernicke’s area (left medial temporal lobe) activity have been observed during performance of memory-encoding tasks [39]. We suggest that the VC, PAC, and SMC activation areas are linked to the neural network associated with PFC during the KPT.

### Prefrontal cortex activity

The reading task revealed relatively little PFC activity. Reading words or sentences is a well trained task and can utilize effective feedback and feedforward neural processing to follow the characters [40]. In contrast, the vowel pick single task generated a great deal of PFC activity. Neural processing was previously reported to be greater for Kana pseudowords compared to Kana words, supporting these results [34]. As shown in Figure 4b, some of prefrontal activation in P did not overlap that in PR. These results suggest that task P required the subjects to utilize alternate neural processing compared to the processing required for picking vowels in task PR.

Within the PFC, bilateral dlPFC activation (left BA 45, 46 and right BA 46) was related to the dual task component of the KPT [25]. This area is highly related to verbal working memory and dual tasks [1,41,42]. One study found only a semantic dual-task condition activated frontal areas, including dlPFC (BA 46) [1]. Kana and English reading experiments comparing semantic and phonological tasks reported left inferior PFC activity (e.g. BA 44, 45, 46, 47) during both types of task [20,30,31,36]. This indicates that this area performs both phonological processing and semantic retrieval. The KPT required subjects to retain content in working memory while picking vowels. Memory storage in three-back tasks have demonstrated greater dlPFC compared to two-back tasks [43]. dlPFC is reported as a brain area that mediates working memory, comprised of short-term storage and executive processes [1]. Based on our results, we propose these components are necessary to execute the KPT.

The majority of imaging studies report primarily left hemispheric activity of the dlPFC during phonological and semantic reading tasks. Both the contrast of PR versus rest and our conjunction analyses found bilateral PFC activity during the KPT. This bilateral activity may be related to updating working memory during the continuous task (reading) of the KPT. Experiments using running span task paradigms reported bilateral activity in BA 9, 10, 11, and 46, suggesting a bilateral network was involved in memory updating [44]. Therefore, our conjunction analyses of bilateral dlPFC may reflect more of the working memory and updating processing rather than that specific to language phonological processing. This bilateral dlPFC finding is more in line with the theory of the dlPFC as an amodal area involved both in multimodal information processing as well as storage [2-4]. The dlPFC, then, is the likely area of the functional bottleneck during the KPT dual task.

Previous investigations of dual task versus single task neural substrates have identified cortical areas that are distinct to dual tasks [41]. Tests of the neural substrates of dual tasks suggest that dlPFC plays a crucial role in the processing required to perform these tasks but activity in this area is not implicated in single tasks (Collette et al., 2005; Jaeggi et al., 2003; Kondo, Osaka, & Osaka, 2004; Low, Leaver, Kramer, Fabiani, & Gratton, 2009; Szameitat, Schubert, Muller, & Von Cramon, 2002). Our research did not substantiate neural activity that was unique to the KPT (PR). However, identification of the dlPFC as the bottleneck region is in agreement with previous findings.

Neural imaging studies specific to the KPT are limited, with some confined to subjects with dlPFC lesions or with Alzheimer’s disease [45-47]. However, lesion and degenerative diseases cannot be construed to equate with neural activity in the healthy brain as compensatory mechanisms may be at work.

### Limitations

This study was only conducted on healthy young adults, therefore we cannot generalize these findings to the healthy elderly or those with MCI. This study focused on healthy young adults because PFC activity in young adults is greater during semantic cognitive tasks compared to older adults.(Logan, Sanders, Snyder, Morris, & Buckner, 2002).

Future studies will repeat this paradigm in healthy older adults and MCI patients. Further studies are also warranted to determine the sensitivity and specificity of the KPT to identify those with MCI. Previous behavioral results of single and dual task paradigms suggest total performance of elderly healthy subjects is better than those of MCI in both single and dual tasks (Montero-Odasso et al., 2009; Pettersson, Olsson, & Wahlund, 2007). This suggests that even single task components (i.e. P or R) might be able to discriminate differences in MCI versus healthy groups. However, to execute the dual task (KPT), more working memory and modified processing in dlPFC is required. Understanding the specifics of PFC activation in the dual task component of KPT (i.e. PR) substantiates that the KPT provides a more sensitive tool allowing for discrimination between groups such as healthy aging and MCI. As shown in this study, we propose BA45 and 46 are specifically being activated in healthy subjects and assume the dual task component in KPT may require processing by this area.

Activation of the dlPFC in the KPT conjuction may be due to task difficulty and not only task specificity. We did not employ an equally difficult task as a contol. However, the complexity hypothesis suggests that dual task costs (computational load) will increase as a function of task complexity. Therefore, complexity cannot be separated from the dual task.

## Conclusion

The KPT is considered to be an effective task for activating the PFC [7]. Direct evidence for a relationship between the test and PFC activity, however, has not been previously reported in a group without neurologic deficit. These results can serve as a baseline to aid in the design and interpretation of future fMRI studies of elderly subjects with and without dementia. The KPT can be applied to languages other than Japanese with relatively simple modifications. We anticipate that results of future studies will provide correlations between PFC activation and performance results for the KPT across different ages. Ultimately, this protocol may be useful for screening for MCI and monitoring cognitive decline associated with dementia and other memory disorders.

## Abbreviations

KPT: Kana Pick-out Test; PFC: Prefrontal cortex; BOLD: Blood oxygenation level-dependent; MCI: Mild cognitive impairment; PR: Picking vowels and reading; P: Picking vowels; R: Reading; EPI: Echo-planar image; FWHM: Width at half maximum; BA: Brodmann's areas; MS/CN: Minimum statistic compared to the conjunction null; SPMs: Statistical parametric maps; SMC: Sensorimotor cortex; PAC: Parietal association cortex; VC: Visual cortex; dlPFC: Dorsolateral prefrontal cortex.

## Competing interests

The authors declare that they have no competing interests.

## Authors’ contributions

AT participated in the design, data collection, and data analyses of the study and helped to draft the manuscript. JAN, SB, YO and YH participated in data analyses and drafting the manuscript. MN supported the development of the experimental procedure with fMRI scans and with KW and MO gave advice for development and analysis of this research project. All authors read and approved the manuscript.
